# Innate Visual Learning through Spontaneous Activity Patterns

**DOI:** 10.1371/journal.pcbi.1000137

**Published:** 2008-08-01

**Authors:** Mark V. Albert, Adam Schnabel, David J. Field

**Affiliations:** 1 Field of Computational Biology, Cornell University, Ithaca, New York, United States of America; 2 Department of Psychology, Cornell University, Ithaca, New York, United States of America; 3 Lincoln Laboratory, Massachusetts Institute of Technology, Lexington, Massachusetts, United States of America; Indiana University, United States of America

## Abstract

Patterns of spontaneous activity in the developing retina, LGN, and cortex are necessary for the proper development of visual cortex. With these patterns intact, the primary visual cortices of many newborn animals develop properties similar to those of the adult cortex but without the training benefit of visual experience. Previous models have demonstrated how V1 responses can be initialized through mechanisms specific to development and prior to visual experience, such as using axonal guidance cues or relying on simple, pairwise correlations on spontaneous activity with additional developmental constraints. We argue that these spontaneous patterns may be better understood as part of an “innate learning” strategy, which learns similarly on activity both before and during visual experience. With an abstraction of spontaneous activity models, we show how the visual system may be able to bootstrap an efficient code for its natural environment prior to external visual experience, and we continue the same refinement strategy upon natural experience. The patterns are generated through simple, local interactions and contain the same relevant statistical properties of retinal waves and hypothesized waves in the LGN and V1. An efficient encoding of these patterns resembles a sparse coding of natural images by producing neurons with localized, oriented, bandpass structure—the same code found in early visual cortical cells. We address the relevance of higher-order statistical properties of spontaneous activity, how this relates to a system that may adapt similarly on activity prior to and during natural experience, and how these concepts ultimately relate to an efficient coding of our natural world.

## Author Summary

Before many animals first open their eyes, neurons in the retina, thalamus, and visual cortex fire spontaneously in highly structured, patterned ways. Experimental manipulations have demonstrated that this activity is necessary for proper function, but it is difficult to answer certain fundamental questions about the role of this activity by using experimental manipulations alone. We know that the early visual system can adapt to better encode statistical regularities in the environment. Can the same learning system that adapts to natural input be applied to this patterned activity to learn the visual code before birth? What qualities would we want in an instructional pattern of activity in the developing visual system? We answer these questions by presenting an abstract model of spontaneous activity in the early visual system—with direct relations to more physiological models. We demonstrate that instructive statistical properties can exist in spontaneously generated patterns based on very simple, local interactions. Also, we demonstrate that these patterns not only have the necessary pairwise correlations, which previous models have relied upon, but also additional sparse, edge-like structure. This higher-order statistical structure is universal to natural visual scenes and is necessary to understand neural responses as an efficient coding of our natural world. Most importantly, this additional structure would allow the visual system to use the same adaptive efficient coding strategy in two cases previously treated as separate—learning from natural visual experience as well as through innately generated patterns before visual experience.

## Introduction

The classic debates of nature vs. nurture, or innate vs. learned, are pervasive in the literature of early visual development. A variety of studies have shown that the visual system requires external experience to mature (e.g., [1]–[3]). On the other hand, many animals are able to see at birth, and have a functioning primary visual cortex even before eye opening (e.g., [4],[5]). It might seem straightforward to assign the properties found at birth to be innate and the properties dependent on visual experience to be learned. However, a strict dichotomy may unnecessarily limit our integrated understanding of visual development. In particular, we wish to focus on the issue of a form of learning that occurs before birth on patterns of activity that are generated internally. It is well known that spontaneous endogenous activity is *necessary*, or permissive, for the proper development of the visual system (see [6] for review). The point of this paper is to discuss the statistical aspects of this activity that may be *sufficient*, or instructive, to guide development in much the same way that visual experience refines the mature visual system. Essentially we propose an “innate learning” approach which prepares the system for later experienced-based refinement – a diplomatic balance between nature and nurture.

**Figure 1:**
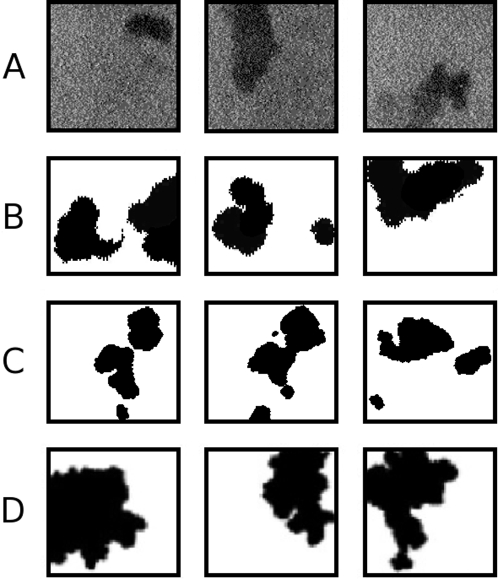
Experimental and theoretical 2D spontaneous activity images. (A) Experimental wave propagation: calcium imaging of a retinal wave (data as described in [9]). (B) Physiological model wave propagation: the ganglion cell layer activation of a retinal wave model (data from model described in [17]). (C) Physiological model wave extent: simulated retinal wave propagated to fullest extent (adapted from [18]). (D) Abstract model wave extent: a pattern generated by the technique used in this paper with parameters (p = 0.55, r = 3, t = 6) as detailed in the methods section.

Several Studies have shown that in the early stages of rat visual development, retinal neurons are spontaneously active and correlated in their bursting patterns of activity [7],[8]. Later, these retinal wave patterns were recorded from many animals by calcium imaging in the developing retina [6],[9], with one example shown in figure 1A. Experiments since then have manipulated these waves by abolishing them, over-stimulating them, or otherwise altering their properties and have shown how they are necessary for proper development [10]–[15]. Several models have been proposed for the production of these waves [16]–[18]. For the two most recent models, cholinergic amacrine cells mediate this activity with general agreement about the mechanism. Neurons begin bursting spontaneously, while neighboring cells can be recruited if enough cells in the local area are also bursting. With such rules for wave formation and propagation, biologically plausible models of retinal wave formation have been able to create complex images, such as those in figure 1B.

Although retinal spontaneous activity has been well studied, many areas beyond the retina exhibit patterned, spontaneous neural activity. In the visual system, both the LGN [19] and V1 [20]–[22] have patterned, spontaneous activity during development. The effects on LGN and V1 connectivity have been analyzed functionally by layer segregation and orientation column formation [23],[24]. Patterned, spontaneous activity is also known to occur in the developing auditory system and is necessary for proper development [25],[26]. Similar developmental mechanisms are also found in hippocampus [27]–[29] and spinal cord [30],[31]. From a biophysical perspective it has been shown that spontaneous neural activity is necessary to mediate many mechanistic effects such as axon branching [32], dendritic patterning [33], and synaptic pruning [23],[34]. With the ubiquity of spontaneous activity in development and its ability to affect various aspects of neural connectivity, understanding the general role of spontaneous activity in early visual development is likely to have implications beyond vision.

**Figure 2:**
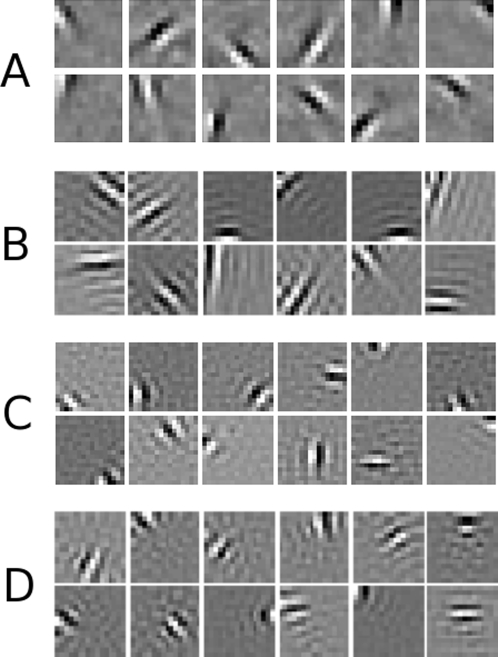
V1 simple cell receptive fields derived through an efficient coding of natural scenes and spontaneous activity patterns. (A) Receptive fields from sparse coding: basis functions derived from natural images (algorithm as described in [40]). (B) Receptive fields from ICA: filters derived from natural images (algorithm as described in [48]). For panels (B,C,D) the same patch collection and efficient coding algorithm was used as detailed in the methods section. (C) Receptive field filters derived from images of simulated retinal waves as in figure 2 of [18] - a few examples are in figure 1c of this paper. Patch size for this data corresponds to approximately 0.3 mm. Refer to the text for the implication of this result. (D) Receptive field filters derived from our generated patterns with parameters (p = 0.7, r = 3, t = 8).

In adult primary visual cortex, it has been known for nearly half a century that V1 cells respond strongly to bars and edges [35] with later experiments demonstrating that simple cells in V1 have a characteristic filter description much like a 2D gabor function [36],[37] as shown in figure 2A. The V1 cell has specific elongated subregions of visual space where relatively bright or dark parts in the visual image will stimulate the cell. Note that this characterization is purely descriptive as a stimulus-response paradigm by answering “what” the neuron responds to instead of “why” the filters have that appearance. According to the efficient coding hypothesis, the role of the early visual system is to remove statistical redundancy in the visual code [38],[39]. From this hypothesis, one way to understand the visual system is to develop and analyze a visual encoding scheme to remove the redundancy in images of natural scenes. This was done using sparse coding [40] and independent components analysis (ICA) [41] on a set of natural images – pictures of rocks, trees, forest scenes, etc... The derived filters resemble the 2D gabor filters found in V1 simple cells – see figure 2B-C. One can conclude from these results that V1 is developed and tuned to efficiently encode the visual world.

In this paper, we make the claim that there is a parsimonious computational reason for the existence of spontaneous patterns - a functional strategy that the early visual system can employ to guide this development both prior to and throughout experience. In addition to molecular guidance cues we believe the visual code is refined from training on patterns of spontaneous activity during development in a similar manner to how the juvenile animals refine the visual code on statistical patterns found in natural images. Many statistical structure models rely on the pairwise correlations between neighboring units (also known as second-order statistics) – an implicit assumption in other functional descriptions of spontaneous activity [42]–[47]. However, many efficient coding models applied to natural images, such as sparse coding and ICA, rely on statistics beyond pairwise correlations. In fact, often as a first step these correlations are removed in a process known as decorrelation or “whitening” (e.g., [40],[41],[48]); a process that at least in part is considered a function of retinal ganglion cells [49] (see [50] for a discussion). Although the developmental activity patterns are known to have relevant pairwise correlations, we argue receptive field refinement may also rely on higher-order statistics – thus bridging the gap between models of sparse, efficient coding and spontaneous activity.

We will demonstrate that simple patterns of activity can be used as training images for refining the visual code. The patterns we use resemble the only 2D imaged spontaneous activity available – retinal waves; this is demonstrated in figure 1, with specific examples of our generated patterns in figure 1D. Beyond a visual resemblance, our pattern generation technique also abstracts from the general properties and parameters of the current retinal wave models. We strongly note, however, that this is strictly not a retinal wave model but an abstraction of what we believe are the essential features of the relevant endogenous activity. We are more concerned in this paper with the statistical nature of the produced activity than its precise localization – including whether the activity originates in one particular area or is part of a larger, dynamical system. For example, in comparison to retinal waves, LGN/V1 spontaneous activity has a more direct influence on cortical receptive field formation. In ferrets the LGN remains spontaneously active at the beginning of V1 activation, while V1 activity and retinal wave production do not significantly overlap in time [6]. LGN and V1 activity have been experimentally characterized [19],[22], but are far less understood than retinal activity, thus prompting our analogies to retinal waves in this paper.

Our patterns are generated using a variant of traditional site percolation models [51] - the analogy to retinal wave propagation and its relation to physiological models is detailed in the discussion section. Models common to the study of critical phenomena in physics, such as percolation models or the Ising model, have been used in artificial neural networks and understanding adult retinal neurons and can be equally useful in understanding models of development. Ising models, for example, have been adapted as artificial neural networks since Hopfield's network [52]. Recent work has also shown that Ising models are apt analogies for the maximal entropy and high-predictability neural firing in the retina upon natural stimulation [53]. Although the pattern generation technique we use is quite abstract, similar networks have been shown to be relevant biologically and demonstrate desirable statistical properties.

The main goal of this paper is to show how the same adaptive, efficient algorithm can be applied for both natural inputs as well as spontaneous activity. We show that certain wavefront-containing patterns possess the relevant statistics and a percolation network provides a useful abstraction for demonstrating this property. These patterns, independent of how they were generated, can simply be used as an existence proof for the possible training role of spontaneous activity. First, we will show our generated patterns qualitatively resemble known patterns of spontaneous activity. We will then compare various methods of learning V1 receptive fields –showing how both natural images and spontaneous activity patterns can be used to produce V1-like gabor filters. We will also demonstrate how significant variations of receptive field properties can occur even at the threshold for scale invariance – showing flexibility of learning even for this simplified model. Finally, one of the main points of this paper, as expressed in the final figure, is that the relevant statistics for sensory coding go beyond simple correlations. There are higher-order statistics which are still present after decorrelation. Sparse and independent efficient coding algorithms rely on these statistics, which are found in natural scenes and are also present in the particular amorphous, wavefront-containing structure of spontaneous activity patterns. We will present how this fact points to the conclusion that the same adaptive coding strategy may then be present both before and during visual experience.

## Results

**Figure 3:**
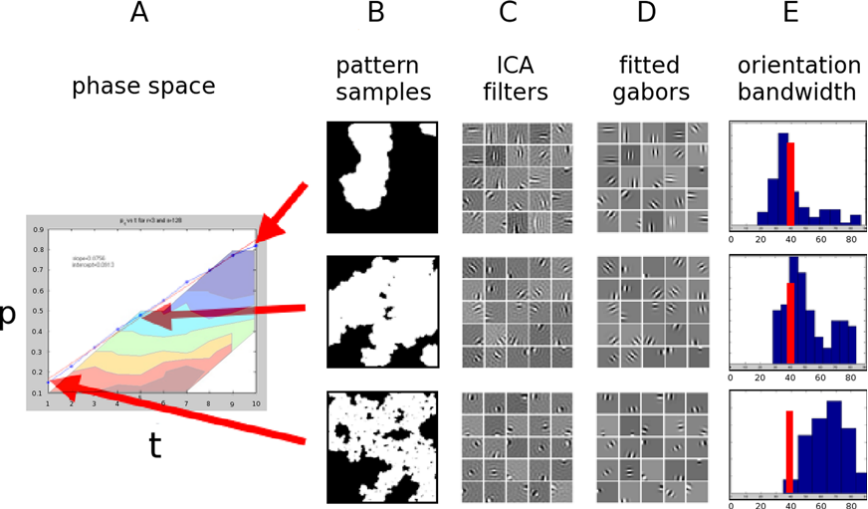
Summary of pattern sampling and analysis demonstrating relevant variation in derived gabor filters. (A) Phase plane with ‘r’ fixed at 3. The curved line in the plot indicates the phase transition boundary as detailed in the methods section. The transparent color contours below the phase transition line indicate the trend for the median orientation bandwidth in that area of the plane. (B) Sampled patterns from (p,r,t) space near the critical percolation threshold - (0.15,3,1), (0.48,3,5) and (0.83,3,10). (C) The 16×16 pixel derived ICA filters. (D) Seven parameter gabor fits of those filters. (E) Histograms of the gabor orientation bandwidths in blue compared to the physiological median in red.

We believe the relevant statistical properties for an efficient “innate learning” strategy are present in a wide class of amorphous, wavefront patterns in which current models of spontaneous activity belong. We hope to demonstrate this generality, and avoid the pitfalls of selecting a particular physiological model, by using an abstract technique for pattern generation. This technique, described in detail in the methods section, can be summarized as a simple, three parameter model – a threshold, site percolation network model. Despite its abstract nature, this technique is analogous to known spontaneous activity patterns in generation and final pattern statistics, as mentioned in the discussion section. We began by exploring the parameter space for a suitable training pattern by varying the proportion of nodes which are able to spread activity, p, and the threshold of active neighboring nodes needed to initiate activity, t. For a fixed t, there is a clear phase transition, the critical percolation threshold, p_c_. For p>p_c_ activity would spread over the whole image, and in the extreme case only a few small areas would remain inactive. At p<p_c_, active clusters would be finite in size, and in the extreme would be exceptionally small clusters – approaching random noise. Although not strictly a property of physiological spontaneous activity patterns, we were interested in scale-invariant patterns in this model. For this reason, sampling was done at p = p_c_ (along the phase transition boundary) as shown in figure 3A. Approximate scale invariance is a property shared with natural images [54]. In this case, it also allows neurons with limited dendritic fields to produce consistent, large-scale statistical effects. We also chose this sampling as a mathematical convenience so results would not require a defined scale of analysis. Note that known spontaneous patterns - such as retinal waves - are clearly not on this self-similar boundary, but may be considered close, with many species having limited wave sizes, and others - such as chick retina - covering large areas of retina and often terminating at the edges [55].

The next step was to find if these patterns could be used to train an efficient coding system for natural vision. Sparse coding and ICA have been used to find approximately independent codes for natural images with resulting filters resembling those found in primary visual cortex, as shown in figure 2A-C. Figure 2C shows the filters derived from natural images given the parameters of image patch collection and coding as detailed in the methods section. Following the main thesis of this paper, one might ask if an efficient coding of activity from more physiologically precise models is capable of producing similar V1-like filters. To show this, we efficiently encoded thresholded, time-lapsed retinal wave images as in figure 2 of Godfrey and Swindale [18]. These moderately resemble images of experimentally determined retinal wave extent as shown in figure 1C (from figure 1 of [56]). The resulting V1-like filters from this data are shown in figure 2D. Although an efficient coding of this model qualitatively produces physiological filters, we would like to demonstrate that these images are embedded in a larger class of amorphous, wavefront-containing patterns capable of producing relevant filter properties. We believe the question of whether or not the activity comes from a particular model - or even from the retina vs. the LGN/V1 - is important, but we would like to stress the necessary statistical properties independent of the particular source. To demonstrate this we generated a set of images from our abstract pattern generation technique with the resulting filters shown in figure 2E for comparison. Note how the general statistical structure of natural scenes, our abstract patterns, and more physiological models of spontaneous activity all produce filters resembling those found in V1.

To further demonstrate the ability of these amorphous, wavefront patterns to generate physiological filters, we generate sets of images along the phase-transition boundary. Filters derived from a representative sample are shown in figure 3C. A qualitative difference between the gabor filters is visible, and we analyzed at least one aspect of these filters – the orientation bandwidth. We chose orientation bandwidth because it is a well-defined, physiologically measured parameter. We fit 7 parameter gabor filters to the 16×16 pixel derived filters. After this fit, we used the parameters of the gabor fits to find the orientation bandwidth, with histograms of these fits shown in figure 3E, along with the primate physiological median [57]. We also coarsely explored the area below the phase transition boundary for this parameter; the transparent color contour in figure 3A indicates how the median orientation bandwidth changes in this region. Note for p<p_c_ a manipulation of ‘p’ is more effective at changing the orientation bandwidth than ‘t’ – one indication of how models such as this one could lead to testable predictions through pharmacological manipulations. However, we do not intend to stress a direct comparisons to physiological filters; we know that even within neurophysiological literature, orientation and spatial frequency bandwidth decreases as newborn macaques age [58] complicating direct comparison. We show that even with this simple generation technique and imposed self-similarity constraint, a significant variation of filters can be produced. This variation demonstrates one way a method like this may adjust local parameters to affect global pattern statistics and more closely resemble properties of adult physiological filters and natural scene efficient coding filters.

**Figure 4:**
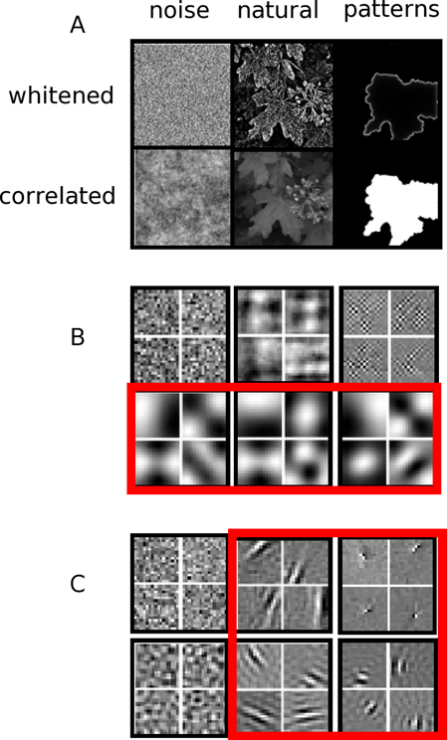
Information relevant to filter formation goes beyond simple, pairwise correlations. (A) Examples of training data for efficient coding. Whitened images were obtained by flattening an assumed 1/f slope in the Fourier amplitude spectrum. (B) PCA bases from each of the six data sets in (A). Note, algorithms which rely on pairwise correlations alone (also known as second order image statistics) only find structure for receptive field formation in correlated data [red rectangle] although much of the useful structure still visible in the decorrelated (“whitened”) images is not captured. Also note the receptive fields in this case are not localized. (C) Representative filters from the same image sets using ICA. Note that the filters from the whitened and unwhitened, natural and wavefront-patterned data qualitatively resemble receptive fields [red square], whereas unstructured 1/f noise does not produce equivalent filters - unlike the results of pairwise correlation-based measures. (note: differences in whitened ICA filter sizes are primarily a product of the 1/f assumption).

Current models proposed to explain pre-experience cortical receptive field development rely primarily on hebbian mechanisms and pairwise correlations. These approaches do not address the relevant statistical structure for receptive field formation related to efficient coding. Although hebbian models are capable of achieving arbitrary levels of complexity - and can even implement sparse coding strategies in specific configurations [59] - we note that the fundamental computational insight of hebbian models relies on pairwise correlations. In figure 4, we address the fundamental differences between these second order and higher order correlations with respect to relevant statistical structure and receptive fields. Note that uncorrelated noise (“white” noise) has no second order or higher order statistics, so techniques that rely on pairwise correlations, as in PCA, or higher-order statistics, as in ICA, do not produce filters with discernable structure. In patterns with only second order correlations, as in the random 1/f patterns (“pink” noise), PCA can produce relevant filters. However, in these 1/f noise patterns the sparse structure on which ICA relies is not present, and structured filters do not form. For the natural and our patterned images in this figure, we have partially removed the second order correlations by a procedure to flatten a 1/f slope in the Fourier amplitude spectrum. This removes the correlations in images that have an approximately 1/f slope, as was shown to be the case for natural images [54]. When we whiten the images by removing the pairwise correlations, PCA bases resembling receptive fields are, by definition, unable to form, and we see that natural images as well as the wavefront patterns still retain important image structure. This whitened structure, reminiscent of line drawings, is efficiently encoded using ICA. Also note that for these image sets the ICA filters are inherently localized within the filter patches. However, encoding using PCA will not produce localized filters without the use of additional constraints. Whichever encoding scheme is used, it should be noted that the generated wavefront patterns have both pairwise correlation structure as well as sparse, edge-like structure used by ICA. If only correlations were necessary to prepare the visual system, there are a number of even simpler ways to create these correlations without the additional wavefront, edge-like structure. This additional, higher-order structure can be exploited by the visual system to guide receptive field formation and maintenance. The fact that it exists in both spontaneous activity patterns and natural scenes suggests that both endogenous and external activity may use the same method of receptive field adaptation.

## Discussion

The point of this paper is to show how seemingly random patterns of activity can be used as training patterns for the visual system before eye opening. We believe that real spontaneous activity patterns are part of a class of amorphous, wavefront-containing patterns with the relevant efficient coding statistics. The patterns we create are also part of this class but abstract out the minimal, essential features while still retaining some biological plausibility. This pattern generation technique is of interest for the following reasons: 1) conceptual and analytical simplicity, 2) statistical properties – both self-similar/correlation-based and sparse coding/edge-like structure, and 3) biological plausibility. First, the technique is a simply stated three parameter model, collapsing to a one parameter model if you fix the neighborhood radius (r = 3, here) and require fractal self-similarity (p = p_c_). Also, this technique is not only conceptually simple, but simple to implement given a biological substrate of dendritic fields, local activity pooling, and activation thresholds. Second, the statistical properties have been discussed in detail – this pattern generation technique is capable of extremes from complete noise to clusters of activity to full activation; self-similar fractal patterns with similar statistics at all spatial scales; and edge statistics which vary the fractal dimension of the edges and consequently the sparse-coding structure of the resultant filters. Third, this technique can be considered an abstraction of more biologically plausible models. The retinal wave model of Butts and Feller [17] showed that wave propagation speed and termination were primarily determined by a 2-D map of one summary variable, f – the local fraction of recruitable amacrine cells – similar to our variable ‘p’. Their random variation of this parameter came from variations in cell refractory period, temporal dynamics from multiple waves, and influence of non-propagating spontaneous activity. Although the more recent Godfrey and Swindale model [18] does not offer an equivalent summary variable, we believe a similar abstraction of local network excitability is equally possible. In the Butts model a neuron would only fire if a threshold of neighboring cells fired, similar to our ‘t’, while in the Godfrey model this threshold varied over time. Both models also had a fixed dendritic field size, analogous to our ‘r’. Their parameters were chosen to match known physiological parameters such as wave size, speed, and frequency given neurophysiological constraints. Our parameters choice, however, was more constrained by theoretical and computational concerns. It may be useful to compare these models; for example, pharmacological manipulations of amacrine cell recruitability or neural firing threshold could move pattern generation along our p-t phase plane vertically or horizontally respectively, leading to potentially testable predictions. We however consider this particular pattern generation technique better suited as a conceptual model to address a developmental paradigm and limitation of current statistical techniques, rather than a guide for directly verifiable experiments. As stated in the introduction, we believe that the use of highly theoretical models such as percolation networks and Ising models have been of sufficient use in understanding neural phenomena [52],[53] to warrant application in this domain.

This method provides an alternative approach to understanding the relation between spontaneous activity and V1 development by stressing the relation to image statistics and efficient coding in individual receptive fields. There are a number of models that stress other physiological dimensions, such as cortical column map formation, which can provide more insights to development. Linsker [42] demonstrated orientation column (OR) formation in a multi-layer model beginning with uncorrelated noise. Grabska-Barwinska and von der Malsburg demonstrate orientation column formation using recent experimental evidence of patchy, spatially periodic cortical spontaneous activity [60]. Miller [45] developed ocular dominance (OD) as well as orientation column (OR) formation. More current models have become even more ambitious in the development of map features. Bednar and Miikkulainen demonstrated direction selectivity (DR) to create a combined map (OR/DR) [61]. A later model combined these features (OC/OR/DR) using translated natural images [44]. Carreira-Perpinan et. al. [46] using the elastic net model [62] included a spatial-frequency map (OC/OR/DR/SF), although the relation of their input to either natural stimulation or spontaneous activity is not clear. In each of these models the goal was to synthesize a cortical map and receptive fields which mimic known neurophysiology. Our use of functional, efficient coding methods precludes any relation to a particular topography, but with this we generate individual receptive fields with properties more relevant to physiological filters – more spatially bandpassed and localized. Our technique also directly addresses how the resulting code reflects its function during natural vision - by similarly efficiently encoding natural inputs using the same adaptive algorithm. Although our model clearly lacks a columnar organization, it does uniquely address the relation of spontaneous activity to current statistical methods of efficient coding.

Although this paper stresses the effects and theoretical justifications of spontaneous activity, there are clearly limitations to this method for preparing V1. Crowley and Katz [24] stated that ocular dominance columns initially form through molecular guidance mechanisms, and subsequent activity was needed for maintenance and plasticity during the critical period. Also, Ringach's connectivity model [63],[64] shows how V1 receptive fields and functional topography could form based on the quasi-regularity of the ON/OFF center retinal ganglion cells in the retinal mosaic; with closely located ON and OFF-center cells forming simple receptive fields. Certainly a number of molecular-guidance mechanisms are necessary for proper development, and even if rudimentary receptive fields can form through simple axon guidance mechanisms, we still believe the simplicity and functional benefits of endogenous activity suggest a plausible role in development. The visual system will eventually refine based on the statistical structure in the experienced natural signals, and the pre-experience receptive fields can refine using the same mechanism on simple patterns. This conceptual model is able to address general properties of this process; however, it is more difficult to address the precise nature of the receptive fields between molecular guidance cues and the onset of natural experience. In addition to physiological details, such as optical and retinal maturity, the goals of this handoff between development processes need to be specified for a given animal. Some precocial animals may require a well functioning visual system from the onset, implying a goal of immediate efficient coding at the expense of later adaptability. On the other hand, altricial animals, such as monkeys and humans, may trade off immediate optimality for a certain amount of environmental adaptability; this may be one functional justification for the large spatial frequency and orientation bandwidths in neonatal monkeys. Although a more detailed, species-specific analysis may require additional assumptions, the general strategy may be universally beneficial. The functional benefits are an increased refinement beyond rough molecular cues using techniques which are relatively simple given the existence of a separate, adaptive learning system.

In summary, our pattern generation technique resembles known patterns of spontaneous activity in both appearance and how they are generated. We have demonstrated that simply-generated, sparse, wavefront-containing patterns have the statistics to produce a sparse, efficient code with filters resembling those found in primary visual cortex and those produced by an efficient coding of natural scenes. Also, this work demonstrates the critical importance of statistics beyond simple pairwise correlations (figure 4) which exist in wavefront-containing patterns. Efficient coding models relating natural scene statistics to V1 activity have relied on higher-order statistics for over a decade. Previous spontaneous activity models that try to explain V1 formation rely only on lower-order statistics that may not be as relevant to early visual processing from a functional perspective. The combination of a simplified abstraction of physiological methods of spontaneous activity and the demonstration that it provides a richer theoretical and computational understanding of why these patterns emerge is clearly attractive as it gives us a better, deeper understanding of the nature of spontaneous activity in development.

With spontaneous activity present in sensory systems, the hippocampus, and motor systems [65], any additional methods of understanding this activity may lead to insights of value in many other areas of brain development. We believe it is useful to add a computational perspective to the mechanistic interpretation of this activity - in addition to the role of spontaneous activity in axon branching, dendritic patterning, and synaptic pruning. Clearly these implementation-level goals are necessary for function, but do not address the general, functional purpose for this connectivity. A statistical, computational perspective is more likely to address the universal and ubiquitous nature of these patterns during development. In this paper, we have given a parsimonious explanation of both why this activity has particular sparse, edge-like statistics beyond simple correlations and how this allows the same adaptive learning system to use both endogenous spontaneous activity and natural inputs to refine the visual code. But more generally, we believe that by examining spontaneous activity in this way, we bring about a conceptual shift in the way people interpret developmental strategies. In the context of the visual system, it appears that the system both learns from patterns extrinsic to its functionality, but strictly internal to the animal; a bridging point between both learned and innate interpretations of mental phenomena.

## Methods

The methods in this paper address the synthesis and analysis of generated activity patterns. The pattern generation technique in this paper was chosen for its theoretical simplicity and its abstraction of essential spontaneous activity features. Its relation to more physiologically detailed models of spontaneous activity [17],[18] is in the discussion section. Details of how the patterns are generated and analyzed are given below.

### Pattern generation

The pattern generation is a variation of site percolation [51]. We use a simple, three parameter (p, r, t) model involving initiation and complete propagation of wave activity – thus the patterns have no temporal component. On a square array of points, mark a random fraction ‘p’ of the points on the grid as potentially active. To initiate an active cluster, we randomly select a location and activate all available points in a neighborhood radius ‘r’. Neighboring potentially active points near the wave can only become active if there are at least ‘t’ active points within a distance ‘r’. The wave is allowed to propagate until no more cells can become active. This completely explains the method of pattern generation, but not the interesting aspects of the behavior.

Introductory percolation theory involves networks with t = 1, often with r = 1 as a typical example. When ‘p’ approaches a value known as the percolation threshold, ‘p_c_’, the pattern of activity is known to be fractal – the image statistics appear similar at all scales. For example, when p<p_c_, the activation terminates forming small clusters, when p>p_c_ the activation spreads without bound leaving small holes without activity, but when p = p_c_, both the activity and holes are nearly infinite in extent – leading to a fractal interpretation. Images at increasingly larger scales become indistinguishable. For examples, the following (p, r, t) triplets are known empirically, and in some cases analytically, to produce fractal images – (∼0.592, 1, 1), (∼0.407, 1.8, 1), (∼0.288, 2, 1) [66]. For our measurements, for a given ‘r’ and ‘t’ pair, we found p_c_ by finding the maximum derivative in the function of cluster size to ‘p’ value. To obtain enough edge statistics on these waves, we randomly chose points to begin wave propagation until more than 20% of the available points were activated – stopping when the last wave was allowed to fully propagate. Only the spatial statistics of these final patterns were explored. All encoding was done by downsampling the image by 2 to minimize any local edge effects due to aliasing. Unless otherwise noted, we set r = 3 for simplicity.

### ICA coding

The method used to analyze these patterns is demonstrated in figure 3. 1) Generate a series of patterns from a given set of parameters, 2) extract image patches from that set, 3) preprocess (“whiten”) the data, and find the optimal code for the data using independent component analysis (details below), 4) fit 2D gabor functions to the resulting filters, and 5) analyze properties of the resulting gabors. We show how they vary with a change in the underlying pattern generation parameters and compare to experimentally measured filters. In this case, we chose orientation bandwidth to demonstrate how filters that qualitatively appear similar can vary in a dimension useful to efficient encoding in the adult.

For both natural images and activity patterns we randomly sampled image patches – excluding patches within a patch width of the border. We also excluded patches without a significant variation for learning – specifically, patches with a pixel variance less than 0.16. This is equivalent to a requirement that between 20%–80% of the original downsampled sites were occupied. For each 256×256 image, downsampled to 128×128, up to 100 viable 16×16 pixel patches were selected. This was done until 10 000 patches were collected, which were then encoded using the fastICA algorithm [48] using the ‘tanh’ contrast function. PCA dimensionality was reduced to 100.
